# Dynamic Response Analysis of Microflow Electrochemical Sensors with Two Types of Elastic Membrane

**DOI:** 10.3390/s16050657

**Published:** 2016-05-09

**Authors:** Qiuzhan Zhou, Chunhui Wang, Yongzhi Chen, Shuozhang Chen, Jun Lin

**Affiliations:** 1 State Key Laboratory of Automotive Simulation and Control, Jilin University, Changchun 130022, China; 2 College of Communication Engineering, Jilin University, Changchun 130022, China; wangch15@mails.jlu.edu.cn (C.W.); chenyz13@mails.jlu.edu.cn (Y.C.); chensz2013@mails.jlu.edu.cn (S.C.); 3 College of Instrumentation and Electrical Engineering, Jilin University, Changchun 130026, China; lin_jun@jlu.edu.cn

**Keywords:** microflow electrochemical sensor, elastic membrane, multiphysical field finite element

## Abstract

The Molecular Electric Transducer (MET), widely applied for vibration measurement, has excellent sensitivity and dynamic response at low frequencies. The elastic membrane in the MET is a significant factor with an obvious effect on the performance of the MET in the low frequency domain and is the focus of this paper. In simulation experiments, the elastic membrane and the reaction cavity of the MET were analysed in a model based on the multiphysics finite element method. Meanwhile, the effects caused by the elastic membrane elements are verified in this paper. With the numerical simulation and practical experiments, a suitable elastic membrane can be designed for different cavity structures. Thus, the MET can exhibit the best dynamic response characteristics to measure the vibration signals. With the new method presented in this paper, it is possible to develop and optimize the characteristics of the MET effectively, and the dynamic characteristics of the MET can be improved in a thorough and systematic manner.

## 1. Introduction

**Figure 1:**
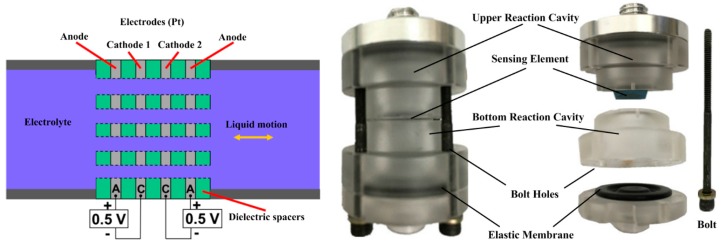
Schematics of the MET (**left**), real product graph and decomposing graph (**right**) structures.

As an important type of sensor widely used in the fields of seismological measurement and geography, acceleration sensors hold a significant position in the marketplace. Among all types of acceleration sensors, acceleration sensors equipped with a solid-mass inertial element account for a high proportion. However, for low or ultralow frequency signal measurement applications, acceleration sensors with a solid-mass inertial element are limited by having a more complex structure, low resistance to interference, higher cost and other shortcomings [1]. Compared to the acceleration sensors equipped with a solid-mass inertial element, a novel microchannel electrochemical sensor (Molecular Electric Transducer, MET) with an electrolytic solution as the liquid-mass inertial element achieves a better performance in the low frequency range field. Electrochemical sensors are used in seismology, seismic exploration, perimeter security systems, structural monitoring [2], among other applications. In the low frequency domain, a MET offers advantages of higher sensitivity, lower cost, high shock resistance and a wider bandwidth. An in-depth study on the use of METs to measure low frequency vibration signals is necessary. Generally, a MET consists of a reaction cavity, an external conditioning circuit and a protective shell. As the core element in the MET, the reaction cavity, filled with electrolyte, with the reversible reaction $I_{3}^{-}+2e^{-}\to 3I^{-},$ ensures the sensing of a vibration signal [3]. In addition, both sides of the reaction cavity are encapsulated by two elastic membranes that influence the dynamic response of the MET. Figure 1 presents the basic concept of the MET and consists of two anode-cathode pairs [1]. The multichannel structure immersed in the electrolyte allows the liquid flow to move inertially, with four electrodes arranged in the order of anode-cathode-cathode-anode (ACCA), which are separated by dielectric layers. The electrolyte is capable of flowing in and out of the multichannel through the electrodes. The usual method to bond the membrane to the reaction cavity is to apply the four-bolt structure shown in Figure 1 (right), thus preventing liquid leakage.

**Figure 2:**
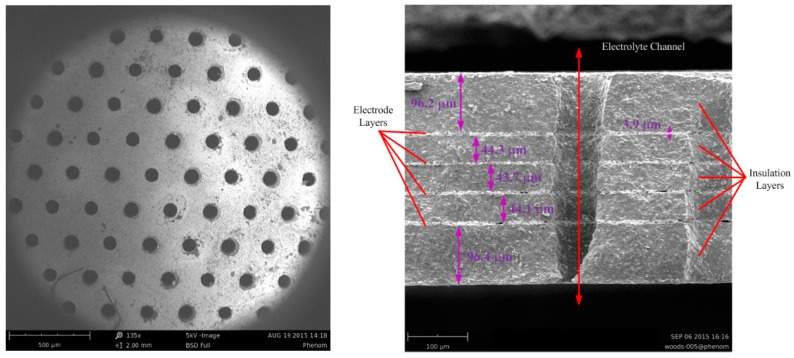
Sensing element with microflow channels (**left**) and dimensions of the intersection of the surface of a single channel (**right**).

**Figure 3:**
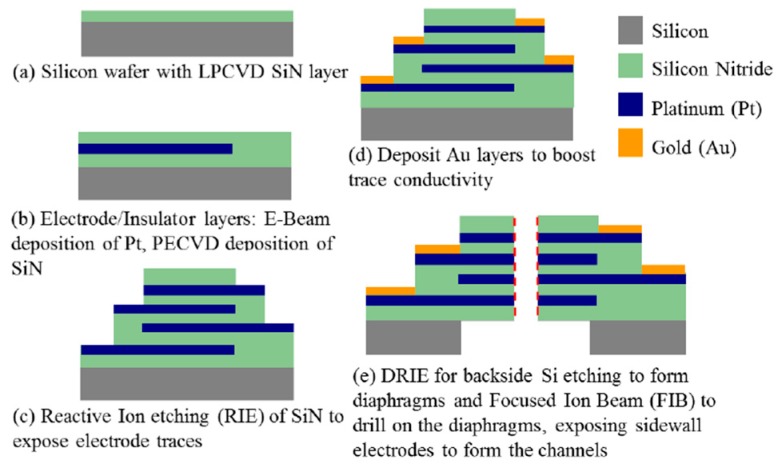
Fabrication process for the sensing element by LTCC technology [1].

A sensing element with microflow channels (Figure 2) is found in the middle of the reaction cavity. Alternately overlapping platinum layers (four) and insulating layers (five) are obtained by a procedure of bonding, pressuring and puncturing by Low Temperature Cofired Ceramic (LTCC) technology (Figure 3).

In the sensing element, due to the fact the external vibration signal and the multichannel are oriented in the same direction, the electrolyte flow changes with the measured vibration signal when the MET is in the working condition. Because the MET is an active sensor, a fixed electric potential difference imposed alternately on the platinum layers is arranged as anode-cathode-cathode-anode (ACCA). With the reversible reaction $I_{3}^{-}+2e^{-}\to 3I^{-}$ on the interface of the cathodes, a miniature electrochemical system is created in the cavity of the MET [1]. When the electrolyte shows ion movement caused by external vibration excitation, a concentration gradient of the electrolyte is generated in the whole reaction cavity, especially on the electrodes in the sensing element. Meanwhile, the enhanced effect of electrode kinetics controls the rate of the electrochemical reaction. Thus, the changing current density derived from the reversible electrochemical reaction on the surface of the electrodes is proportional to the signal from the external vibration. Thus, the differential current generated by two cathodes, which is the source of the MET output, can be the output from the cathodes after amplification by the conditioning circuit because the interface charge transfer is associated with the generation and absorption of the tri-iodide ions on the electrode surface. Therefore, the electrical current through any electrode can be related to the flux of active ions towards or away from the electrode, according to the following equation:

(1) $$I=\text{Dq}\left(\overset{}{\underset{S}{\oint }}\left(\nabla c,n\right)\right)dS$$

where D is the diffusion coefficient, c is the concentration of the active charge carriers, q is the charge transferred across the interface in single electrochemical reaction, **n** is a unit vector normal to the surface of the electrode, and integration is performed over S, the electrode surface area.

The operating principle of MET can be described as follows: when an electric voltage is applied to the electrodes, an electrochemical (background) current appears. When an external vibration appears, the electrolyte starts moving due to inertia, and the convective transport of ions changes the electrode current according to the mechanism described above. The difference in the cathode currents in the two anode-cathode pairs is used as the MET output signal. Although each electrode current is non-linear with respect to the fluid velocity, the combined output of both cathodes is linear for a very wide range of fluid velocities. The MET output current is given by [3]:

(2) $$I_{out}=I_{2}-I_{1}=\text{Dq}\left(\overset{}{\underset{S2}{\oint }}\left(\nabla c,n\right)\right)dS2-\text{Dq}\left(\overset{}{\underset{S1}{\oint }}\left(\nabla c,n\right)\right)dS1$$

where $\;I_{2}$ and $I_{1}$ are the currents through the surface of the corresponding cathodes, and $S_{2}$ and $S_{1}$ are the surface areas of the corresponding cathodes.

The optimization of the design of the MET requires too many physical parameters of the complex electrochemical process to be obtained directly from practical measurement. Under practical conditions, due to the different materials, manufacturing processes, experimental conditions and other issues, many variables such as flow field distribution in the reaction cavity, a variety of charging ion concentration gradients and the electrode surface ion concentration, *etc.*, cannot always be measured directly by practical experiments, so a few problems of the MET cannot be solved by traditional methods.

Recently, with the development of numerical simulation technology, multiphysics finite element analysis methods can process the joint simulation of a complex electrochemical and microfluidic model. Combined with the traditional methods, the use of numerical simulation aids in the development of a comprehensive analysis of the study of METs. Since 2010, many numerical simulation models have provided effective solutions in the relevant MET study fields. Because the study of the MET combines electrochemical, electrical, microfluidic and other complex multiphysics aspects, this study takes advantage of the current new simulation technology methods to accomplish the modelling of the study object. With the numerical simulation, physical characteristics that could not be measured and observed before in the practical experiments can be studied effectively. Beginning in 2012, based on numerical simulation and practical experiments, Sun [4,5], Vadim [6,7,8] and Huang [1,9,10] have studied the theory and the complex electrochemical changes in the MET reaction cavity. By combining the results of practical experiments with numerical simulations, a number of performance optimizations can be accomplished by analysing the actual results measured by data acquisition equipment and the simulations that involve effective concentrations of charged ions and comprehensive velocity-field distributions.

Compared with traditional acceleration sensors, its special structure and measurement principles make the MET an effective transition from a solid inertial element to a liquid inertial element due to a great innovation. With its special inertial element, unique advantages for vibration signal measurement in the low frequency domain can be achieved. For the MET, new methods have become available to guarantee its measurement accuracy.

First, the elastic membrane element in the MET causes the electrolyte to be encapsulated in the reaction cavity. When an external excitation signal arrives, the liquid inertial element not only generates a movement in the same direction as the vibration direction but also generates a movement in the perpendicular direction, and a trend in the flow is followed. Under normal conditions, the electrolyte should remain in a state of standard laminar flow in the reaction cavity. However, due to the kinetic energy caused by the high frequency or large amplitude of the external vibration signal, a slip happens between the layers of the electrolyte that is exposed to laminar flow. When the electrolyte is moving, to prevent the electrolyte from generating a series of slips between the laminar layers, the elastic membranes that are positioned on both ends of the reaction cavity are capable of maintaining the laminar flow of the electrolyte under various forms of external vibration motion. Only if the laminar flow state is maintained, will the MET be operated under the best performance conditions.

According to the theory mentioned above, the elastic force generated by the elastic membrane can control the movement of the electrolyte in the reaction cavity. When an external vibration occurs, the electrolyte in the reaction cavity of the MET moves in the same way as the vibration signal. Thus, the electrochemical current signal that acts as the source of the output of the MET is proportional to the vibration intensity on the cathodes. The elastic membrane element has the main function of adjusting the electrolyte motion. However, for different measurement fields, the design of the elastic membrane influences the dynamic response of the MET. To compensate and decrease the negative impact caused by improper design, in this paper, a novel elastic membrane design method that can adapt to the different structures of the MET is proposed. Combining the practical size of the MET with numerical modelling, the state of movement of the electrolyte in the reaction cavity and elastic membranes with different thicknesses can be observed by the multiphysics finite element method. With the same pulse signal imposed on two METs with different elastic membranes, a series of evaluations can be accomplished. The optimal design of the elastic membrane can be calculated by comparing variables such as the response time. In addition, in this paper, relevant practical experiments are also applied to verify the correctness of the numerical simulation. Finally, amplitude-frequency and phase-frequency characteristics are discussed to describe the influence of different membranes on the MET.

## 2. Mathematical Modelling

**Figure 4:**
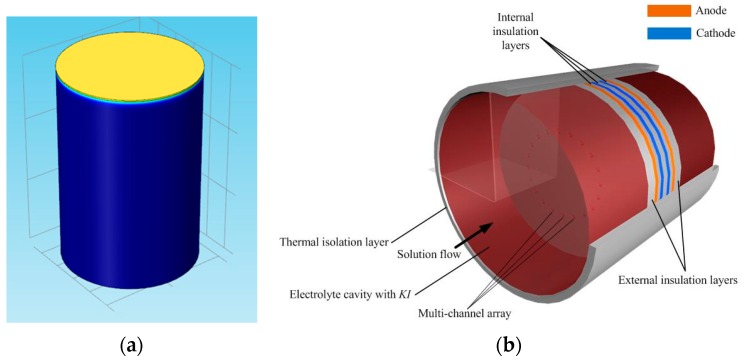
The structure of the reaction cavity (**a**) and the elastic membranes in the numerical simulation and the structure inside the reaction cavity (**b**).

The geometric structure of the MET is built as shown in Figure 4 (left). The elastic membrane element (yellow) is placed on the top of the cavity. The reaction cavity is marked as blue. The inside structure is presented in Figure 4 (right). The membrane element is on the top of the numerical model. In the simulation, a Gaussian pulse response signal is used as the external vibration excitation. By observing the movement state of the electrolyte in the reaction cavity and the elastic membrane, the dynamic response characteristics of the membrane can be calculated by the numerical simulation.

The numerical simulation parameters of the dimensions and materials are shown in Table 1.

Navier-Stokes equations are applied to build the fluid dynamic model of the electrolyte as follows:

(3) ∇v=0

(4) $$\frac{\partial v}{\partial t}=-\frac{\nabla P}{\rho }+\frac{\mu }{\rho }\nabla^{2}v+a$$

where ***v*** and *t* denote the velocity of the electrolyte flow and time. The electrolyte density is $\rho =1.473\times 10^{3}\mathrm{kg}/m$. *P,* a scalar, represents the pressure, and *μ* = 1.4 × 10^–3^ Pa is the dynamic viscosity. Parameter ***a*** is the acceleration of the external signal.

In this study, Gauss’s law is applied to demonstrate charge conservation:

(5) $$-\nabla \cdot (\varepsilon \nabla \varphi )=\rho^{\prime }$$

where ε, φ and $\rho^{\prime }$ are the permittivity (F·m^–1^), potential (V) and charge density (C·m^–3^), respectively.

In addition, the Nernst-Plank-Poisson equation is used to describe the mass transport (Equations (6) and (7)) subject to mass continuity (Equation (8)). Because of the high degree of nonlinearity, multiple length and time scales are observed, and the applicable approximation, which applies to scales larger than nanometres, is the assumption of electroneutrality (Equation (7)) [11]:

(6) $${\vec{N}}_{i}=-D_{i}\nabla c_{i}-z_{i}m_{i}c_{i}\nabla \varphi +c_{i}\vec{u}$$

(7) $$c_{K^{+}}=c_{I_{3}^{-}}+c_{I^{-}}$$

(8) $$\frac{\partial c_{i}}{\partial t}+\nabla \cdot {\vec{N}}_{i}=R_{i}=0$$

where ${\vec{N}}_{i}$ is the flux of species *i* (mol·m^–2^·s^–1^) and *D_i_* is the diffusion coefficient of species *i* (m^2^·s^–1^). Here we set *D*_*I*^–^_ = *D*_*K*^+^_ = 2.8 × 10^–9^ m^2^·s^–1^ and *D*_*I*_3_^–^_ = 2.0 × 10^–9^ m^2^·s^–1^. *c_i_* is the concentration of species *i* (mol·m^–3^), *z_i_* is the charge number of species *i*; *m_i_* is the mobility of species *i* (m^2^·V^–1^·s^–1^), $\vec{u}=[u,v,w]$ is the velocity vector (m·s^–1^), and *R_i_* is the mass source of species *i* (mol·m^–3^·s^–1^).

Assuming electroneutrality and small absolute concentration gradients of charge-carrying electrolyte species, the electrolyte current obeys Ohm’s law (Equation (9)) subject to a constant conductivity (Equation (10)). Meanwhile, the concentration of the electrolyte is effectively infinite, Fick’s 2nd law for an incompressible flow is applied (Equation (11)) as follows:

(9) −∇⋅(σ∇φ)=Q

(10) $$\sigma \approx F\sum_{i}z_{i}^{2}m_{i}c_{i}$$

(11) $$\frac{\partial c_{i}}{\partial t}=\nabla^{2}c_{i}-\vec{u}\cdot \nabla c_{i}$$

where σ is the conductivity (S·m^–1^), Q is the charge source (A·m^–3^·), and F is the Faraday constant.

Combining the equations from Equation (6) to Equation (11), the partial differential equations (PDEs) are applied in the Comsol Multiphysics software as shown below [3]:

(12) $$\frac{\partial c_{I^{-}}}{\partial t}+\frac{\partial c_{I^{-}}u}{\partial x}+\frac{\partial c_{I^{-}}v}{\partial y}+\frac{\partial c_{I^{-}}w}{\partial z}=-m_{I^{-}}F\left[\nabla c_{I^{-}}\cdot \nabla \varphi +c_{I^{-}}\nabla^{2}\varphi \right]+D_{I^{-}}\nabla^{2}c_{I^{-}}$$

(13) $$\frac{\partial c_{I_{3}^{-}}}{\partial t}+\frac{\partial c_{I_{3}^{-}}u}{\partial x}+\frac{\partial c_{I_{3}^{-}}v}{\partial y}+\frac{\partial c_{I_{3}^{-}}w}{\partial z}=-m_{I_{3}^{-}}F\left[\nabla c_{I_{3}^{-}}\cdot \nabla \varphi +c_{I_{3}^{-}}\nabla^{2}\varphi \right]+D_{I_{3}^{-}}\nabla^{2}c_{I_{3}^{-}}$$

(14) $$\frac{\partial c_{K^{+}}}{\partial t}+\frac{\partial c_{K^{+}}u}{\partial x}+\frac{\partial c_{K^{+}}v}{\partial y}+\frac{\partial c_{K^{+}}w}{\partial z}=m_{K^{+}}F\left[\nabla c_{K^{+}}\cdot \nabla \varphi +c_{K^{+}}\nabla^{2}\varphi \right]+D_{K^{+}}\nabla^{2}c_{K^{+}}$$

In addition, the Butler-Volmer condition is set up to convey the procedure of electrode kinetics:

(15) $$2\vec{n}\cdot {\vec{N}}_{I_{3}^{-}}=-\frac{2\vec{n}\cdot {\vec{N}}_{I^{-}}}{3}=-2k_{a}c_{I_{3}^{-}}e^{\left(-\alpha F/RT)(U-\varphi -E_{0}\right)}+k_{c}c_{I^{-}}e^{\left(1-\alpha )(F/RT)(U-\varphi -E_{0}\right)}\alpha$$

where *n* is the out-normal of the electrode surface, and $k_{a}=k_{c}=4\times 10^{-9}\;m^{2}/s$ are the anodic and cathodic reaction constants, respectively. The parameter α is the charge transfer coefficient for the cathodic reaction and is set to 0.5 in this study. U = 0.8 V is the imposed electric potential at the electrodes, and E_0_ = 0.54 V is the equilibrium potential.

Instead of applying the shell interface, which is barely coupled with the hydrodynamic model of the electrolyte in the cavity, extensive efforts were made to build a suitable solid-liquid interface, which was inspired by tracking the immiscible fluid-fluid interface. The membrane model offered by the structural mechanics of Comsol Multiphysics can be coupled with the modified Navier-Stokes equations, including the surface tension force [11,12]. To be the input to the stress load interface of the membrane model, the surface tension force is imposed on the membrane in the vertical direction. According to the relationship between stress and train yields to the constraints such as linear elastic condition and solid material setting, the force distribution on the membrane in the traverse direction is also capable of being calculated. As a feedback effect from the membrane to the electrolyte, the movement state of the electrolyte in the reaction cavity can be influenced, corresponding to the movement state of the membrane. Therefore, variables such as velocity and force distribution for both electrolyte and membrane can be solved by the finite element analysis method.

The topology of the solid-liquid interface changes with time for the contact interface of the solid elastic membrane and the electrolyte. The level set method as well as the phase field method are both well suited for modelling moving boundaries where topology changes occur [13]. Both methods are available in the CFD module and the structure mechanics model. In this solid-liquid two phase model, the level set function determines the solid-fluid interface by tracing the isolines of the level set function, *Φ*. The equation governing the transport and initialization of *Φ* is:

(16) $$\frac{\partial \Phi }{\partial t}+u\cdot \nabla \Phi =\gamma \nabla \cdot \left(\varepsilon \nabla \Phi -\Phi \left(1-\Phi \right)\frac{\nabla \Phi }{\left|\nabla \Phi \right|}\right)$$

where **u** is the fluid velocity, and γ (m/s) and ε (m) are the initialization parameters. The ε parameter determines the thickness of the membrane around the interface where *Φ* goes from zero to one. When stabilization is used for the level set equation, we can typically use an interface with a thickness of ε = *h_c_*/2 to describe the initial membrane, where *h_c_* is the characteristic mesh size in the region encompassed by the solid-liquid interface. A suitable value for γ is the magnitude of the maximum velocity occurring in the model. Because the level set function is a smooth step function, it is also used to determine the density and dynamic viscosity globally by:

(17) $$\begin{array}{l} \rho =\rho_{l}+(\rho_{m}-\rho_{l})\Phi \\ \mu =\mu_{l}+(\mu_{m}-\mu_{l})\Phi \end{array}$$

Here *ρ_l_*, *μ_l_*, *ρ_m_* and *μ_m_* denote the constant density and viscosity of the electrolyte and the membrane, respectively [11]:

(18) $$\begin{array}{c} \rho \left(\frac{\partial u}{\partial t}+u\cdot \nabla u\right)=-\nabla p+\nabla \cdot \mu \left(\nabla u+\nabla u^{T}\right)+\rho g+F_{st}\\ \nabla \cdot u=0 \end{array}$$

In Equation (18), *ρ* represents the density (kg/m^3^) of the electrolyte, and **u** is the velocity (m/s). The parameters *t* and *p* are time (s) and pressure (Pa), respectively. The momentum equations contain gravity, *ρ*g, and the surface tension force is also denoted by $F_{st}$.

The surface tension force in Equation (16) $F_{st}$ is defined by:

(19) $$F_{st}=\nabla \cdot T=\nabla \cdot \left\{\sigma \left[I+\left(-nn^{T}\right)\right]\delta \right\}$$

where σ is the surface tension coefficient, **I** is the identity matrix, **n** is the interface unit normal, and δ is a Dirac delta function. The interface normal is calculated from:

(20) $$n=\frac{\nabla \Phi }{\left|\nabla \Phi \right|}$$

The level set parameter Φ is also used to approximate the delta function by a smooth function defined by [13]:

(21) δ=6|Φ(1−Φ)||∇Φ|

when the surface tension force $F_{st}$ is confirmed by using the stress and strain interface of the membrane model, the surface tension force is proportional to the stress in the vertical direction. Constrained by the material setting and the relationship between stress and strain in the membrane model, the elastic force generated in the transverse direction is also calculated.

The constraints and boundary conditions are described as follows: the bottom of the cavity uses no-slip conditions, **u** = 0. The sidewall of the cavity surrounding the electrolyte uses the wetted wall as its boundary condition. The centre axis of the cylinder cavity corresponds to the symmetry boundary condition. For the membrane constraints, the outer edge of the membrane is supported in the transverse direction and the edge connecting the membrane with the sidewall is set as a fixed constraint. To summize the modelling process, a modified Navier-Stokes equation based on the principles of a two phase flow is presented to describe the state of the movement of the electrolyte. Meanwhile, the surface tension force at the solid-liquid surface is derived by the modified Navier-Stokes equation. To strongly couple the liquid phase with the solid phase, the surface tension force acts as the input of the stress force in the membrane model. Due to the elastic material setting and the stress-strain relationship of a membrane structure, the force distribution on the membrane will be solved integrally. As the feedback from the solid phase to liquid phase, the force and velocity change would correspondingly affect the movement state of the electrolyte. With the constraints and the boundary conditions mentioned above, the level set function is applied to describe the solid-liquid interface. Coupling the membrane with the electrolyte tightly also makes effective communication between the electrolyte and the membrane by the variable velocity.

## 3. Analysis and Discussion

**Figure 5:**
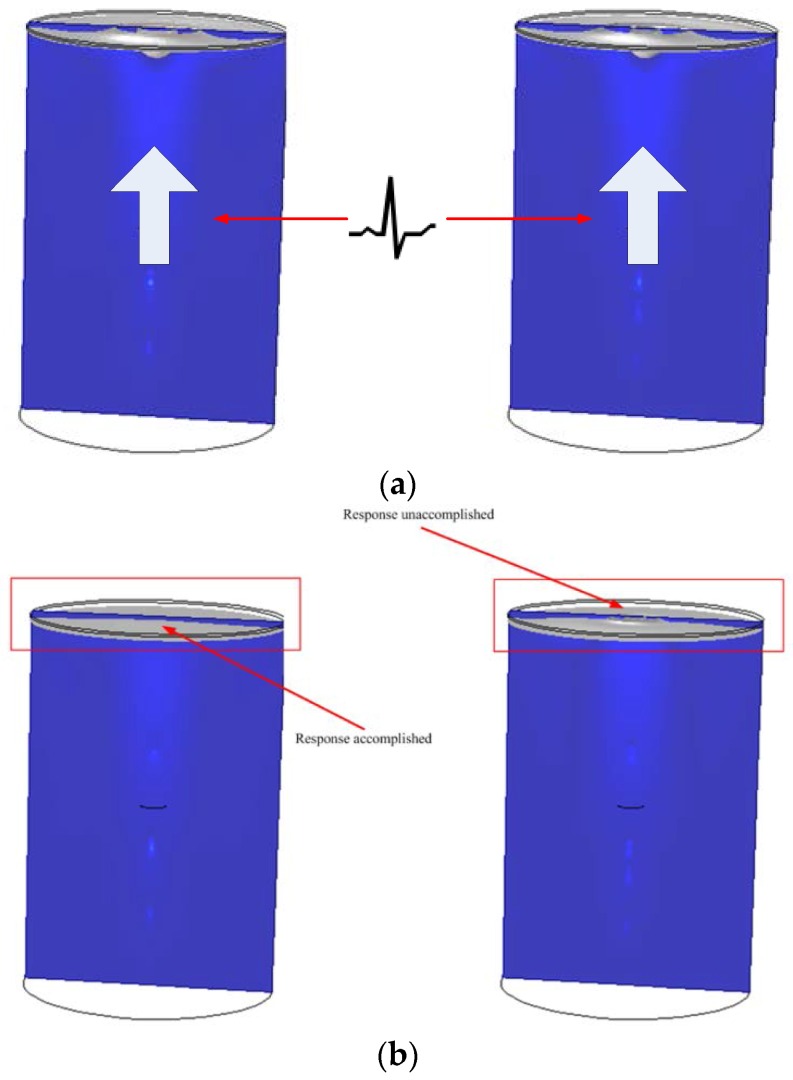
The performance of the different membrane thicknesses under the same Gaussian pulse signal excitation. (**a**) 0–0.3 s, the same pulse signals are imposed on the 1.5 mm thick membrane (**left**) and on the 1.0 mm thick membrane (**right**); (**b**) 0.3–0.5 s, the response of the reaction cavity with a membrane thickness of 1.0 mm is not determined.

To compare the effect of using different elastic membrane elements, the same parameters of the MET are adopted in this paper. Two thickness types of elastic membrane with the same reaction cavity are simulated in the numerical experiments. In the numerical simulation experiments, the same Gaussian pulse signals of acceleration y = exp(–((x − 0.3)/0.2)^2)) are applied in the models as the external excitation signals. By observing the movement of the elastic membranes, elastic membranes that use different thicknesses can be evaluated. The result of the numerical simulation is shown in Figure 5. The same pulse signals are imposed on the reaction cavities that only have different elastic membranes.

**Figure 6:**
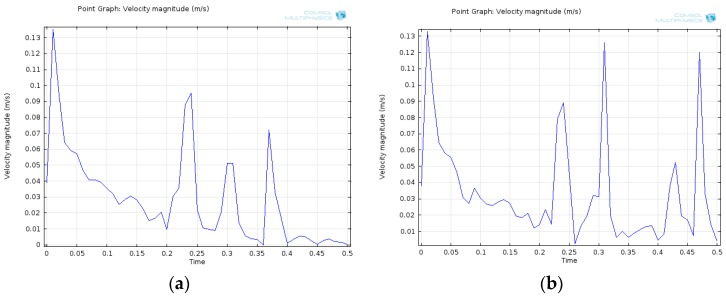
Time (s) *vs.* velocity magnitude (m/s) of the MET with a membrane thickness of 1.5 mm (**a**) and a membrane thickness of 1.0 mm (**b**).

In the beginning, the electrolyte is accelerated by the same pulse signal. Due to the energy loss, the amplitude of the moving membranes is reduced, and the state of the electrolyte tends to be stable. After 0.5 s, the MET with the membrane thickness of 1.5 mm regresses to the stable state and waits for the arrival of the next signal. However, because of the 1.0 mm membrane thickness, the other MET is still responding to the pulse signal at 0.5 s. From the result of the numerical simulation, the MET with 1.0 mm thick membrane is not capable of reducing the energy in a short time. To observe the result of the numerical simulation, a probe is inserted on the centre of the membrane to acquire the magnitude of the moving velocity of the membrane at all times. From the probe’s observations, the movement state of the membrane is shown with the specific curves in Figure 6.

In Figure 6, at 0 s, the two METs give similar pulse signals with the same magnitude. From 0 to 0.5 s, the MET equipped with a 1.5 mm membrane effectively restrains the electrolyte and helps the liquid inertia regress to the stable state. Meanwhile, the 1.0 mm membrane affects the strength of the constraint. The MET with a 1.0 mm thick membrane is more suitable for the measurement of long-periodic vibration signals. Above all, by using numerical simulation, different elastic membrane models can be built by the multiphysics field element method. The performance and the effect of the membranes can also be evaluated in this way.

## 4. Results and Discussion

**Figure 7:**
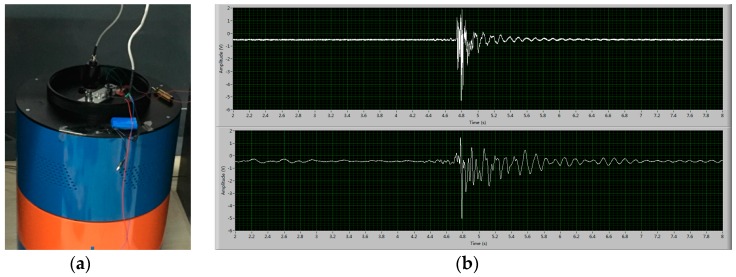
Experimental environment of the vibration table and the data acquired from NI PXI (a product of National Instrument Corporation).

To support the numerical simulation results, practical experiments comparing different membrane structures were also applied in this paper. The experimental parameters of the materials and the structure and dimensions for membrane are listed in Table 2. Figure 7 shows the experimental setup and the recorded signals.

**Figure 8:**
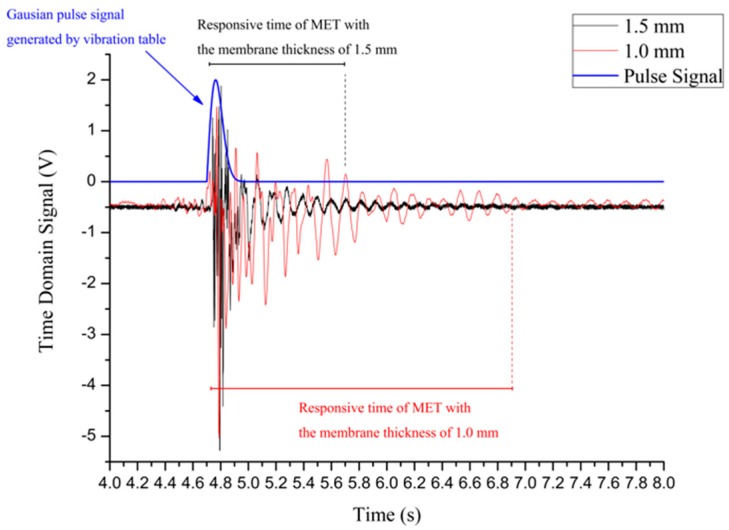
Practical experiment for the MET with different elastic membranes.

By using the NI PXI data acquisition equipment the results of the practical experiment for two METs with different membrane thicknesses are presented in Figure 8.

By analysing the results shown in Figure 8, at the beginning, the Gaussian pulse signals marked in blue produce an effective external vibration excitation with the same amplitude for both METs. The MET with the 1.0 mm membrane a longer response time. The MET with a 1.5 mm membrane can regress to the stable state in a shorter time.

**Figure 9:**
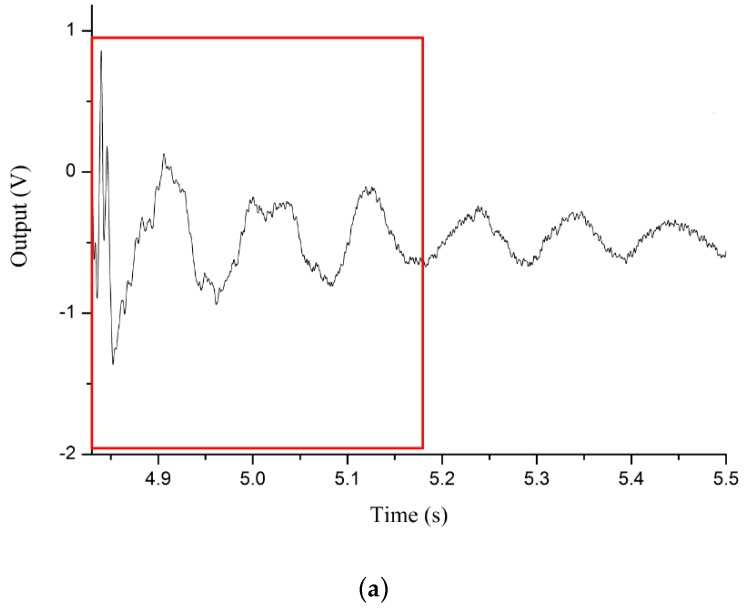

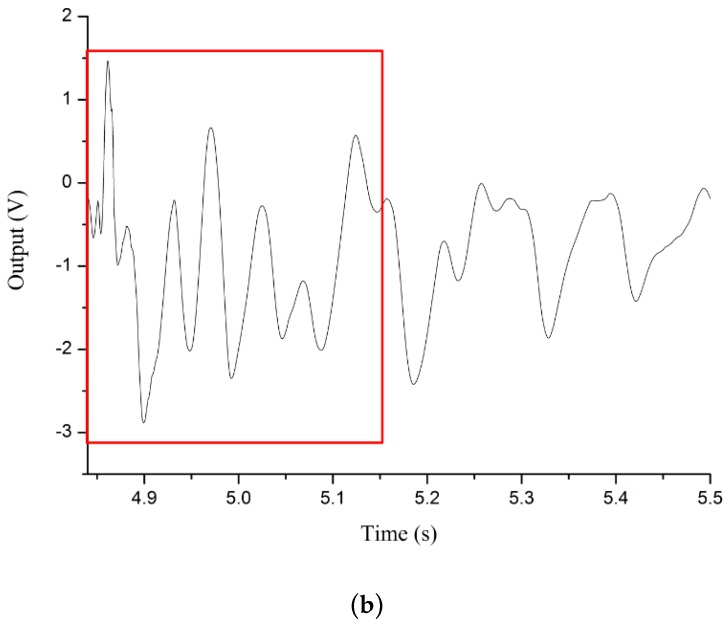
Segments captured from Figure 8 corresponding to the simulation results in Figure 6, and (**a**) is the result of practical experiment for MET with 1.5mm membrane thickness corresponding to the simulation in Figure 6a; (**b**) is the result of practical experiment for MET with 1.0mm membrane thickness corresponding to the simulation in Figure 6b.

To discuss the correspondence between modelling and the experimental data, a segment captured from the simulation results in Figure 6 is compared with the experimental result in Figure 9. First, comparing both the left figures in Figure 6 and Figure 9, the MET with the 1.5 mm membrane is activated by an external pulse signal, and the first peak appears in both left figures simultaneously. The peaks following this highest peak dampen gradually, and the curves in the practical experiment convey the same trend. The curves of the right figures in both Figure 6 and Figure 9 share the same trend and shape. Therefore, this practical experiment consisting of a pulse signal response test conveys that the results meet the expectations of the numerical simulation in Figure 6, and the result of the practical experiment in Figure 9 is presented to verify the numerical simulation.

However, for the dynamic response characteristics, the pulse signal is insufficient to completely reveal the dynamic response characteristics of the MET with different types of membranes. Another practical experiment was studied to investigate the influence of different elastic membranes.

**Figure 10:**
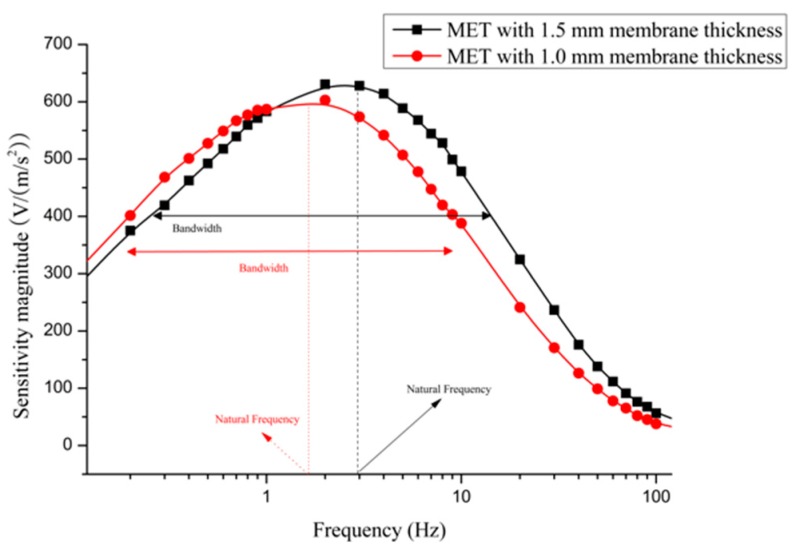
Frequency *vs.* sensitivity magnitude of the MET that is equipped with different membrane thicknesses.

The sensitivity-frequency curves for two types of MET equipped with both membranes are shown in Figure 10. From the distribution of the points, the dynamic response for the MET can be represented by the result of the practical experiments. In Figure 10, the MET with the 1.5 mm thick membrane has a higher natural frequency. The natural frequency as a significant parameter affects the sensitivity in the low frequency domain. For a MET that has a low natural frequency, the capacity to detect the low-frequency signal will be much better. The MET with a 1.0 mm thick membrane has a lower natural frequency, so this type of membrane is more suitable for a MET that requires better measurement performance in the low frequency range. However, from the bandwidth point of view, the MET with the 1.0 mm membrane covers a shorter range in the frequency domain. Compared with the 1.5 mm membrane, the MET with the 1.0 membrane always has a narrow band range but a stronger capacity to measure low frequency signals. From the practical experiment data in Figure 10, the amplitude-frequency characteristics are revealed to describe the dynamic response of the MET.

**Figure 11:**
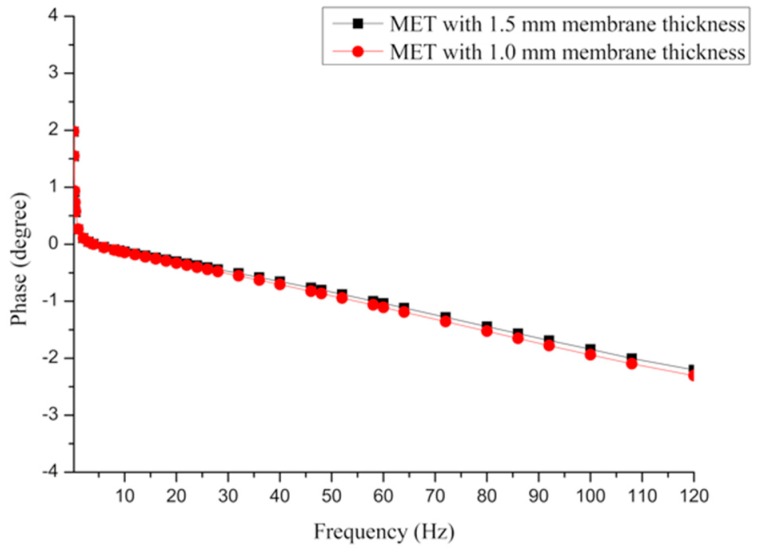
Frequency *vs.* phase of the MET that is equipped with different membrane thicknesses.

Moreover, to completely study the dynamic response of the MET affected by different types of membranes, another practical experiment involving the phase-frequency characteristics is necessary, and the results are shown in Figure 11. By comparing two curves from the aspect of phase-frequency, the MET with the 1.5 mm membrane can maintain a little phase shift in the frequency domain. Thus, analysis of the dynamic response for the METs with two types of membrane is explained, and the influence of the elastic membrane is also presented above.

## 5. Conclusions

The elastic membrane element, a significant element in METs, is the main focus of this paper. From the numerical simulation experiments based on the multiphysical field finite method the elastic membrane and the cavity of the MET are studied integrally. Meanwhile, the relationship between different elastic membrane elements and the dynamic response of the MET are derived in this paper. Combining the numerical simulation with practical experiments, the elastic membrane can be customized for several of vibration measurement fields. Thus, the adaptation mentioned above can ensure the best dynamic response characteristics for the MET.

## Figures and Tables

**Table 1:** Numerical simulation parameters.

Membrane Material	Low Density Polyethylene (LDPE)
Membrane Radius	r = 1.25 cm
Membrane Thickness	h_1_ = 1.0 mm, h_2_ = 1.5 mm
Young’s Modulus	E = 0.2 GPa
Poisson’s Ratio	V = 0.4
Mass Density	*ρ* = 900 kg/m^3^
Cavity Height	H = 4.0 cm
Cavity Material	Polymeric Methyl Methacrylate (PMMA)
Cavity Radius	R = 1.2 cm
Multi-channel Centre Distribution Radius	R_centre_ = 0.13 cm

**Table 2:** Experimental parameters.

Membrane Material	Low Density Polyethylene (LDPE)
Membrane Radius	r = 1.25 cm
Membrane Thickness	h_1_ = 1.0 mm, h_2_ = 1.5 mm
Membrane Density	*ρ* = 900 kg/m^3^
Cavity Height	H = 4.0 cm
Cavity Material	Polymeric methyl methacrylate (PMMA)
Cavity Radius	R = 1.2 cm
Shape of the Membrane	Circular
Structure of the Sensing Element	Four-electrode structure with circular hole by LTCC fabrication
